# Electrical Impedance Tomography as a monitoring tool during weaning from mechanical ventilation: an observational study during the spontaneous breathing trial

**DOI:** 10.1186/s12931-024-02801-6

**Published:** 2024-04-25

**Authors:** Jantine J. Wisse, Tom G. Goos, Annemijn H. Jonkman, Peter Somhorst, Irwin K. M. Reiss, Henrik Endeman, Diederik Gommers

**Affiliations:** 1 Department of Adult Intensive Care, Erasmus Medical Centre, Rotterdam, the Netherlands; 2 Department of Neonatal and Pediatric Intensive Care, Erasmus Medical Centre – Sophia Children’s Hospital, Rotterdam, The Netherlands; 3 Department of Biomechanical Engineering, Faculty of Mechanical, Maritime and Materials Engineering, Delft University of Technology, Delft, The Netherlands

**Keywords:** Electrical Impedance Tomography, EIT, Spontaneous breathing trial, Weaning

## Abstract

**Background:**

Prolonged weaning from mechanical ventilation is associated with poor clinical outcome. Therefore, choosing the right moment for weaning and extubation is essential. Electrical Impedance Tomography (EIT) is a promising innovative lung monitoring technique, but its role in supporting weaning decisions is yet uncertain. We aimed to evaluate physiological trends during a T-piece spontaneous breathing trail (SBT) as measured with EIT and the relation between EIT parameters and SBT success or failure.

**Methods:**

This is an observational study in which twenty-four adult patients receiving mechanical ventilation performed an SBT. EIT monitoring was performed around the SBT. Multiple EIT parameters including the end-expiratory lung impedance (EELI), delta Tidal Impedance (ΔZ), Global Inhomogeneity index (GI), Rapid Shallow Breathing Index (RSBI_EIT_), Respiratory Rate (RR_EIT_) and Minute Ventilation (MV_EIT_) were computed on a breath-by-breath basis from stable tidal breathing periods.

**Results:**

EELI values dropped after the start of the SBT (*p* < 0.001) and did not recover to baseline after restarting mechanical ventilation. The ΔZ dropped (*p* < 0.001) but restored to baseline within seconds after restarting mechanical ventilation. Five patients failed the SBT, the GI (*p* = 0.01) and transcutaneous CO_2_ (*p* < 0.001) values significantly increased during the SBT in patients who failed the SBT compared to patients with a successful SBT.

**Conclusion:**

EIT has the potential to assess changes in ventilation distribution and quantify the inhomogeneity of the lungs during the SBT. High lung inhomogeneity was found during SBT failure. Insight into physiological trends for the individual patient can be obtained with EIT during weaning from mechanical ventilation, but its role in predicting weaning failure requires further study.

**Supplementary Information:**

The online version contains supplementary material available at 10.1186/s12931-024-02801-6.

## Background

Mechanical ventilation is one of the cornerstones in the treatment of intensive care patients. Invasive mechanical ventilation is live saving, but may cause lung injury. Prolonged weaning from mechanical ventilation is associated with increased mortality [1, 2]. Therefore, weaning and liberation from mechanical ventilation should be pursued from the initiation of invasive ventilation and choosing the right moment for extubation is essential [3–6]. A test that is widely used and integrated in clinical practice to guide clinicians in choosing the right moment for extubation is the spontaneous breathing trial (SBT). During the SBT, the patient is challenged to sustain adequate ventilation and oxygenation while the amount of support given by the ventilator is reduced or stopped. Along with the result of the SBT, the health care professional decides if the patient is ready for extubation [6]. Given the central role of weaning tests in extubation decisions, understanding their physiology is essential. However, few studies have investigated this essential point [4], likely due to the lack of simple to use and validated lung monitoring techniques.

Electrical Impedance Tomography (EIT) is a noninvasive imaging modality and a promising tool to gain physiological insight into the weaning process. EIT estimates the distribution of air inside the lungs based on changes in electrical impedance [7–9]. A belt integrated with electrodes is placed around the thorax, and small iterating currents are injected into the belt and measured between the electrodes [8]. EIT provides a dynamic image of the distribution of air in the lungs [10]. Multiple parameters can be extracted from the EIT signal providing information about the tidal volume, functional residual capacity, the center of ventilation, the (in)homogeneity of the ventilation and the percentage of collapse. Previous studies [11–14] performed with EIT around the SBT found two correlations: first, a relation between lower end-expiratory lung impedance (EELI) levels and SBT failure [11, 12] and second, a higher global inhomogeneity (GI) index was related to SBT failure [13]. A recent study investigating EIT parameters around extubation found that 2 h after extubation the GI index was higher in patients that failed extubation. Before and after extubation the amount of ventilated pixels was lower in patients that failed extubation [15].

This physiological study was conducted to determine whether EIT parameters during long term EIT measurements surrounding a T-piece weaning trial are potential predictors for SBT failure. We addressed two important topics: first, a more detailed evolution of previously described [11–15] and new EIT parameters during the SBT and second, the relation between EIT parameters and SBT success or failure. Last, we propose a novel combination of EIT preprocessing techniques to enhance reliability of long term EIT measurements.

## Methods

### Study design and population

This observational physiological study was conducted in the Intensive Care Unit of the Erasmus Medical Center, Rotterdam, The Netherlands. Adults who were invasively ventilated for at least 24 h and considered ready for an SBT within the next 48 h were considered for enrollment. The protocol was approved by the local ethics review committee (MEC-2020–0521). Written informed consent was obtained from either the subject or their legal representative. Exclusion criteria were contraindications (thoracic wounds, pneumothorax, internal pacemaker or defibrillator) for an EIT measurement or transfer to another hospital before extubation.

### Study protocol

Continuous EIT monitoring was performed with the EIT belt (LuMon™ Belt, Sentec AG, Therwil, Switzerland) applied between the 4th and 6th intercostal space. EIT data was obtained with a sampling frequency of 50.2 Hz. Ventilator data were automatically logged with a sample frequency of 1 Hz. Continuous Transcutaneous CO_2_ (TcCO_2_) was obtained using a dedicated sensor (SenTec OxiVent, Sentec AG, Therwil, Switzerland) applied to the skin. Measurements were conducted prior to and during the SBT and were continued until 24 h after extubation.

A T-piece SBT was performed according to the local clinical protocol. The protocolled length of the SBT was 30 min. Objective criteria for SBT failure were PaO_2_ ≤ 8 kPa or SaO2 < 90% with FiO_2_ ≥ 40%, pH < 7.32, respiratory rate > 35 breaths/min or an increase in respiratory rate ≥ 50%, systolic hypertension > 180 mmHg or an increase in systolic blood pressure > 20%, systolic hypotension < 90 mmHg, and arrhythmia. Subjective criteria for SBT failure were fear and anxiety, low consciousness, heavy transpiration, cyanosis, and use of accessory respiratory muscles. Weaning failure was defined as a failed SBT or reintubation with 48 h after extubation.

### EIT data preprocessing and analysis

Data preprocessing and analysis were performed in MATLAB R2021a (Mathworks, Natick, USA). First, an EIT preprocessing method was designed and applied to remove outliers, artifacts and noise components, which included automated selection of periods with stable tidal breathing (see Supplemental materials and figure A1 [16–18]). During offline processing it was noted that the reliability of prolonged EIT measurements was limited, hampering adequate comparison of post-extubation EIT signals with those measured around the SBT (see Discussion section). Therefore, data analysis was only performed for stable measurement periods just before, during and directly after the SBT.

As the duration of the SBT trial varied from patient to patient, each SBT was divided into 5 equal stable time intervals (epochs). The number of epochs was equal for every patient but could vary in length. Data were analyzed on a breath-by-breath basis and were then averaged per epoch. The following parameters were quantified with EIT:

- delta Tidal Impedance (ΔZ)
- end-expiratory lung impedance (EELI)
- respiratory rate (RR_EIT_)
- minute volume (MV_EIT_); with tidal volume derived from ΔZ using a point calibration between the tidal volume (ml) as measured by the mechanical ventilator and the ΔZ [AU] from EIT. The calibration factor was individualized for each patient, and one or an average of multiple calibration points were chosen from stable breathing periods before the SBT.
- rapid shallow breathing index (RSBI_EIT_); based on the calibration between mechanical ventilation tidal volume and ΔZ.
- center of ventilation (CoV)
- global inhomogeneity index (GI) [19], describing the homogeneity of ventilation distribution, calculated as the sum of the absolute difference between the median value of ΔZ per pixel divided by the sum of all impedance values [19]:
  1 $$GI=\frac{\sum_{x,y\in lung}\left|{DI}_{xy}-Median\;\;({DI}_{lung})\right|}{\sum_{x,y\in lung}{DI}_{xy}}$$

Only pixels within the lung contour were included for this calculation. We used predefined lung contours in the LuMon^TM^ software. Sentec selects thorax and lung models adapted to the individual patient from a set of CT-derived thorax and lung models. A GI of zero represents a perfect homogenous ventilation distribution; the larger the GI, the more inhomogeneous the tidal volume distribution within the lung area.

- Nondependent and dependent silent spaces (NSS, DSS), reflecting poorly/non-ventilated lung areas. Pixels within the lung contour with impedance changes < 10% of the maximum impedance change were quantified as silent space. Pixels below or above the CoV were considered dependent or nondependent silent spaces, respectively.
- Functional lung space (FLS), quantified as 100% minus the percentage of pixels defined as silent space.

### Statistical analysis

Statistical analysis was performed using R version 2022.07.2 (RStudio, Posit Software, Boston MA, USA). Descriptive data are presented as mean $\pm$ standard deviation; the distribution of normality was checked through the Shapiro -Wilk test. Repeated measurements were compared with linear mixed-effects models with fixed effects of measurement step and random effect of subject; estimated means were compared after Tukey correction. This model was extended with fixed effect of group (to test the difference between SBT success and failure) and group by measurement step interaction to test for their interaction effect (i.e., to test if the change in repeated measurements was different between the group with successful SBT and failed SBT). Appropriate transformations were applied for those parameters in which the mixed model residuals did not fit a normal distribution. For all analyses, *p*-value < 0.05 was considered statistically significant.

## Results

### Study participants

A total of 23 patients ready for weaning were enrolled, their characteristics are presented in Table 1. Five patients failed the SBT. At baseline, the SAPSII score was higher for the patients who failed the SBT (36 vs. 53). The total ventilation duration (12 vs. 26 days), the ICU length of stay (15 vs 32 days) were significantly higher for the SBT failure subgroup (*p* < 0.05 for all). The PaO_2_ after the SBT (68 vs 103 mmHg) was significantly lower for the SBT failure subgroup and the length of the SBT was shorter in patients that failed the SBT.

**Table 1:** Baseline characteristics in the overall population and success and failure SBT subgroups

	Total Population (*n* = 23)	Successful SBT (*n* = 18)	Failed SBT (*n* = 5)	*p*-value
Gender (M/F)	M:14, F:9	M:12, F:6	M:2 F:3	
BMI (kg/m^2^)	27.8 (5.8)	26.7 (5.3)	31.6 (6.6)	0.1
Age (years)	63 [57.5 70]	62.5 [57.2 67.2]	70 [69 73]	0.26
SAPS II	39.8 (16.6)	36.2 (13.3)	52.8 (22.3)	< 0.05
SOFA at study enrolment	4.9 (2.6)	4.9 (2.9)	4.8 (1.3)	0.95
Days ventilated before study (days)	11.3 (5.8)	10.7 (5.9)	13.4 (5.0)	0.37
Total ventilation duration (days)	15.3 (8.5)	12.4 (5.9)	25.6 (8.7)	< 0.05
Pressure support ventilation before SBT (days)	3 [2 7]	3.5 [2 7]	3 [2 6]	0.51
SBT length (min)	31 [24.5 35.5]	33 [30.2 35.8]	15 [9 21]	< 0.05
ICU length-of-stay (days)	19.3 (10.7)	15.7 (7.4)	32.3 (11.8)	< 0.05
ICU mortality	2	1	1	
Admission diagnosis (n, %)				
Respiratory	10 (43%)	7 (39%)	3 (60%)	
Neurological	6 (26%)	6 (33%)	0 (0%)	
Cardiovascular	3 (13%)	2 (11%)	1 (20%)	
Trauma	3 (13%)	2 (11%)	1 (20%)	
Sepsis	1 (4%)	1 (6%)	0 (0%)	
Respiratory mechanics at study baseline (clinical settings)				
Total PEEP (cmH_2_O)	7.5 (2.1)	7.3 (1.8)	8.4 (3.2)	0.50
dP (cmH_2_O)	8.1 (3.2)	7.8 (3.2)	9.2 (3.5)	0.44
PaO_2_ before the SBT (mmHg)	84.8 (20.3)	86.3 (21.8)	79.6 (15.8)	0.45
PaO_2_ after the SBT (mmHg)	95.3 (36.8)	102.8 (36.8)	68.3 (23.3)	< 0.05
PaCO_2_ before the SBT (mmHg)	41.3 (5.3)	41.3 (6.0)	42.0 (3.8)	0.66
PaCO_2_ after the SBT (mmHg)	40.5 (6.0)	39.8 (5.3)	42.8 (9.0)	0.49
RSBI before the SBT	40.1 [32.1 53.9]	37.8 [31.5 48.8]	53.7 [53.3 59.5]	0.18
P/F ratio before the SBT	232 [205.5 303.5]	231 [207 318]	233 [188 279]	0.39
FiO_2_ (%)	35 [30 40]	35 [30 38.8]	35 [35 40]	0.48

**Fig. 1:**
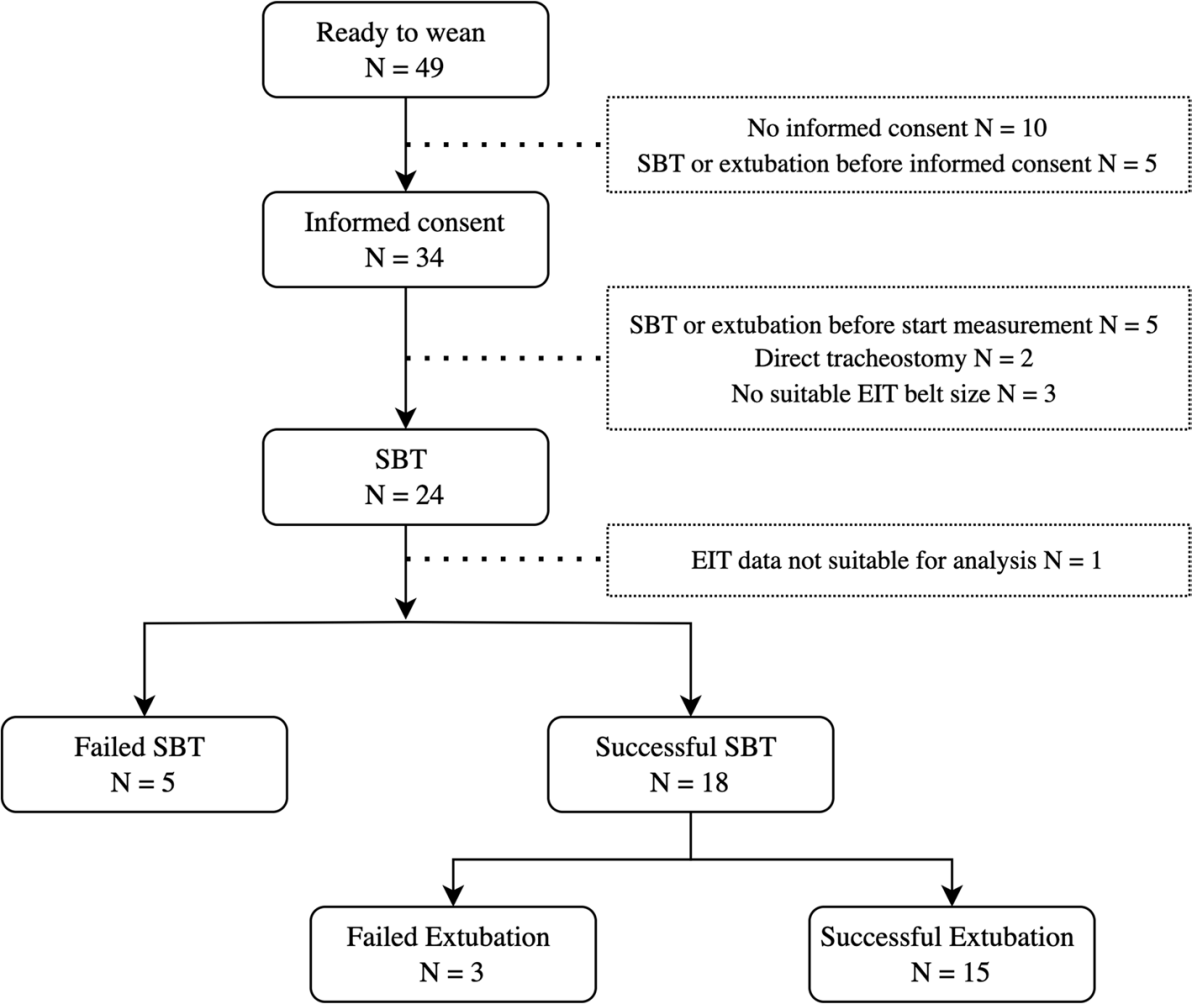
The flow chart summarized the weaning process from the study participants from ready to wean to extubation

Figure 1 summarizes the weaning process of the study participants. Three patients were reintubated within 48 h after extubation, while 15 patients were successfully weaned. From the overall population, five patients were extubated directly after the SBT without restarting mechanical ventilation between the SBT and extubation.

### EIT trends during the SBT for the whole group

**Fig. 2:**
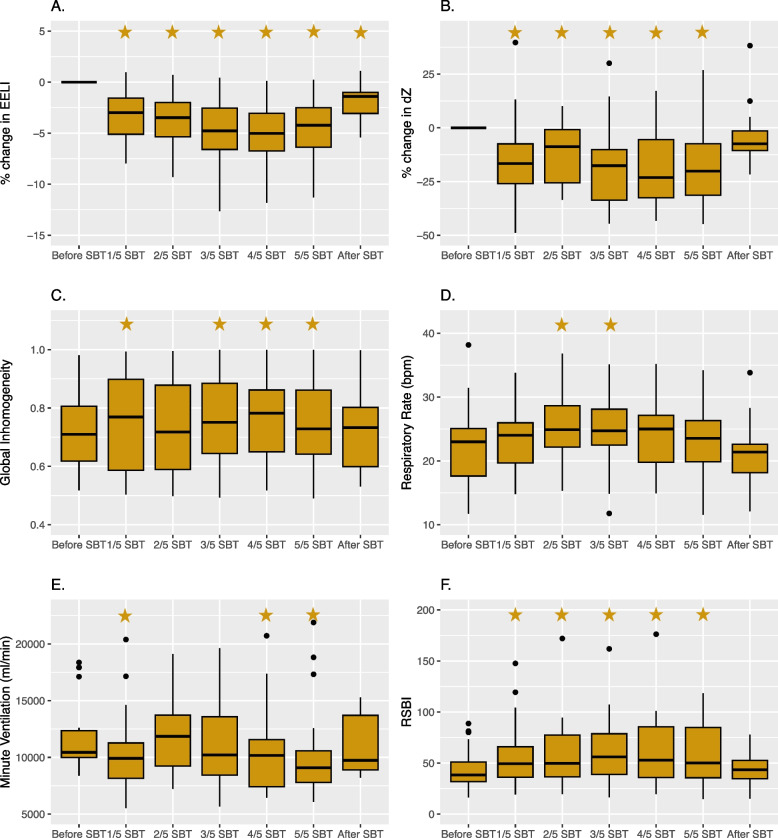
EIT parameters trends during the SBT extracted from stable tidal breathing periods before the SBT at 1/5th, 2/5th, 3/5th, 4/5th, 5/5th and after the SBT. **A** Percentual change of EELI during the SBT. **B** Percentual change of delta tidal impedance (ΔZ) during the SBT. **C** The Global Inhomogeneity (GI) during the SBT. **D** Respiratory Rate (RR_EIT_) during the SBT. **E** Minute ventilation (MV_EIT_) during the SBT. **F** Rapid shallow breathing Index (RSBI_EIT_) during the SBT. – Significant difference from the baseline measurement is indicated with a star

EIT parameters during the SBT for the whole group are presented in Fig. 2. The EELI was decreased throughout the SBT, reflecting a drop in end-expiratory lung volume (Fig. 2a). After restarting mechanical ventilation, EELI did not recover to its baseline value. The delta tidal impedance (ΔZ) was decreased throughout the SBT (Fig. 2b) and quickly restored to its initial value after ventilator reconnection (within an average of 24 s). The GI increased after initiation of the SBT (Fig. 2c) and recovered to its initial value after restarting mechanical ventilation.

Additional trends in EIT-derived parameters are a significant increase in the RR_EIT_ (Fig. 2d) and a significant decrease in the MV_EIT_ (Fig. 2e) halfway through the SBT. Both the MV_EIT_ and RR_EIT_ were restored to their baseline values after mechanical ventilation was restarted. The RSBI_EIT_ (Fig. 2e) increased significantly after the start of the SBT and recovered to its baseline value after restarting the mechanical ventilation.

The other EIT parameters, including NSS, DSS, FLS, left–right and ventral-dorsal ventilation distribution, and the center of ventilation (COVx, COVy), did not show any significant trend from baseline (see Supplemental Figure A2).

### SBT failure and success group

**Fig. 3:**
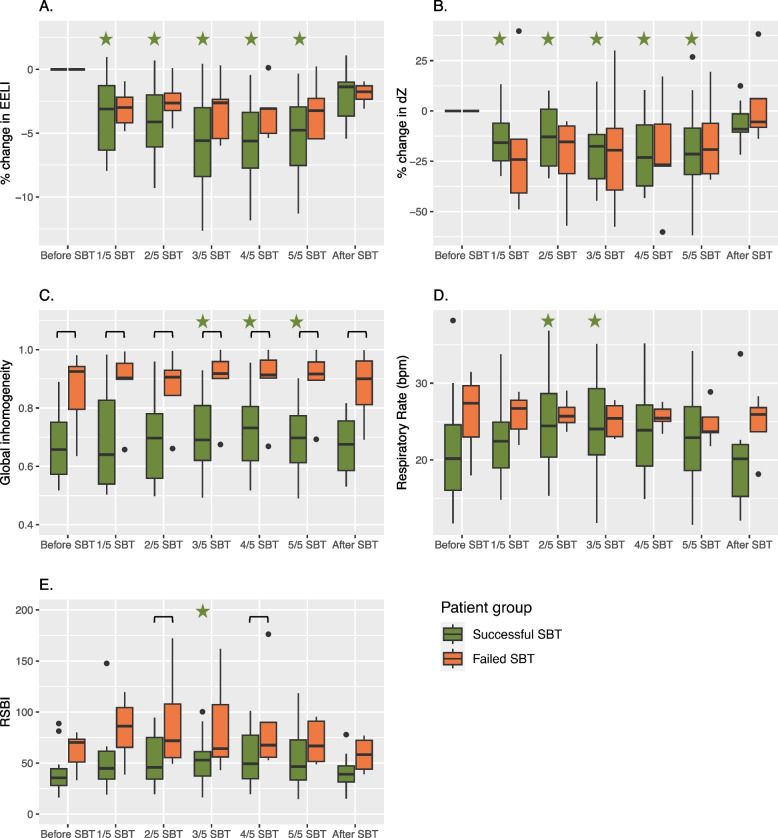
EIT parameters trends during the SBT extracted from stable tidal breathing periods before the SBT at 1/5th, 2/5th, 3/5th, 4/5th, 5/5th and after the SBT. The group is divided between patients with a successful SBT (green) and a failed SBT (orange). **A** Percentual change in the EELI during the SBT. **B** Percentual change of delta tidal impedance (ΔZ) during the SBT. **C** The Global Inhomogeneity (GI) during the SBT. **D** RR_EIT_ during the SBT. **E** Rapid shallow breathing Index (RSBI_EIT_) during the SBT. – Significant differences (*p* < 0.05) between the two groups are indicated with a horizontal bar (—﻿), significant differences from the baseline measurement (*p* < 0.05) successful SBT is indicated with a green star. Significant differences from the baseline measurement (*p* < 0.05) for the failed SBT is indicated with an orange star

Differences between patients who failed (*n* = 5) or successfully passed (*n* = 18) the SBT are presented in Fig. 3. The percentual change in EELI and ΔZ were not significantly different between the two groups. No significant differences in EELI and ΔZ were found during the course of the SBT within the failure group. Therefore, the whole group changes in EELI and ΔZ (Fig. 2a and b) were mainly driven by the successful SBT patients. The GI (Fig. 2c) was significantly different between the two groups throughout all measurements, with higher GI values in patients who failed the SBT. Within this group, GI remained stable during the SBT, while an increasing GI was found for the success group.

Another significant difference between the two groups in EIT parameters was an increase in the RR_EIT_ halfway during the SBT in the success group. The RSBI_EIT_ was significantly different between the two groups at 2/5 and 4/5 of the SBT. The other EIT parameters including MV_EIT_, FLS, NSS, DSS, left–right and ventral-dorsal ventilation distribution, COVx and COVy, did not show any significant changes per group or between the two groups (see Supplemental figure A3).

We explored the difference in EIT parameters between patients with a successful SBT and successful extubation and a successful SBT and reintubation. No differences in these parameters were found between the two groups but analysis is limited by the small sample size of only three patients failing extubation (supplemental figure A4).

### Transcutaneous CO_2_ measurements

**Fig. 4:**
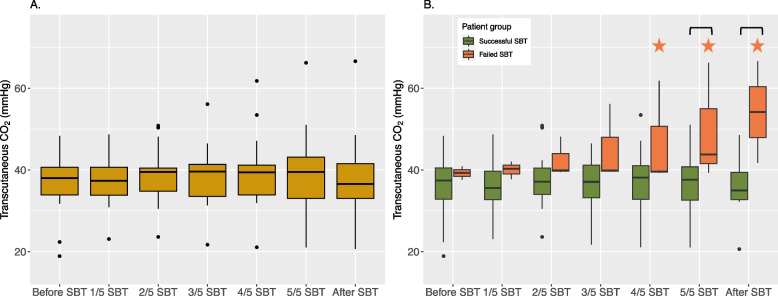
**A** Transcutaneous CO_2_ values of all patients during the SBT. **B** Transcutaneous CO_2_ measurements divided between patients with a successful SBT (green) and a failed SBT (orange). – Significant differences (*p* < 0.05) between the two groups are indicated with a horizontal bar —, significant differences from the baseline measurement (*p* < 0.05) successful SBT is indicated with a green star. Significant differences from the baseline measurement (*p* < 0.05) for the failed SBT is indicated with an orange star

The TcCO_2_ trend (Fig. 4A) did not show any significant effects over time. However, there were significant differences between the success and failure groups (Fig. 4B): the TcCO_2_ was stable during the SBT in the success group, but increased rapidly during the SBT in the failure group.

## Discussion

This study provides detailed insights into the respiratory physiology during a T-piece trial as assessed by EIT and synchronized with transcutaneous CO_2_ measurements. EIT parameters provide trend information of the ventilation distribution during the SBT, and findings can be summarized as follows: 1.) multiple EIT parameters showed significant changes from baseline during the SBT. The EELI, ΔZ and MV_EIT_ dropped after the start of the SBT. The respiratory rate, GI and RSBI_EIT_ increased during the SBT. After restarting mechanical ventilation, EELI did not recover to its baseline value. The other investigated EIT parameters: FLS, NSS, DSS, COV and transcutaneous CO_2_ did not significantly change from baseline during the SBT. 2.) Two parameters were significantly different between patients with a successful and failed SBT. First, the GI was significantly higher at all time points in patients failing the SBT. Second, the transcutaneous CO_2_ was significantly higher at the end of the SBT in patients who failed the SBT compared to patient who successfully passed the SBT.

In addition to previous studies, we observed lung derecruitment after the SBT, as EELI values after restarting the mechanical ventilation were significantly lower than prior to the SBT. However, note that changes were only small and therefore the clinical relevance of these findings can be questioned. In contrast to EELI, the ΔZ immediately recovered to baseline, within seconds after restarting the mechanical ventilation The combination of these results suggests derecruitment by a drop in EELI without loss of tidal volume, and that lung recruitment after resuming mechanical ventilation is a slower process. This supports the idea that resuming mechanical ventilation after a successful SBT and prior to extubation may be beneficial [20]. The drop in EELI results partially from PEEP loss during the T-piece SBT. Divergent outcomes can be anticipated during pressure-support ventilation (PSV) SBT where PEEP is (partially) retained and work of breathing is lower [12, 21]. It is also important to realize that the drop in EELI may not solely be related to reduced ventilation, but can also be caused by fluid-related changes in thoracic impedance. We did not screen for weaning induced pulmonary oedema during the SBT [22] and could therefore not discriminate between the two potential causes of reduced impedance measurements.

We found the GI index to be significantly different between those patients with a successful SBT and those with a failed SBT. The GI index for patients failing the SBT was higher compared to patients with a successful SBT, and this difference already existed prior to the SBT. These results are in accordance with previous literature where the GI was described as a potential identifier for patients at risk for weaning failure, but no clear cutoff values were proposed [11, 23, 24]. GI values were found higher after extubation in patients with extubation failure, however the GI index was not significantly different before extubation. Jousselin et al. argue that mechanical ventilation before extubation could help for homogenization of the distribution of the ventilation [15]. However, we found higher GI values before the start of the SBT on pressure support ventilation in patients failing the SBT. We explored the difference in GI after extubation in our cohort. No differences in these parameters were found between patients with successful or failed extubation but analysis is limited by the small sample size of only three patients failing extubation (supplemental figure A5). The GI is a feasible index to summarize the complex pulmonary impedance distribution pattern into a single number [25]. A higher GI implies more regional differences in lung mechanics, which could be caused by e.g., ventilation-perfusion mismatch, atelectasis or inflammation. The role of GI in predicting patients at risk for SBT failure, including optimal cutoff values, requires further study.

The RSBI has been used as a predictor of weaning success where Yan and Tobin proposed that patients with an RSBI of < 105 were more likely to be successfully extubated [26, 27]. We found RSBI values < 105 throughout the SBT in all but one patient, meaning that this threshold was not discriminative for predicting weaning success in our cohort. However, it should be noted that the RSBI was calculated from ΔZ converted to tidal volumes instead of tidal volumes (ml). ΔZ derived tidal volumes were calculated by utilizing the relationship between the two during the pre-SBT period and applying a one point calibration [28]. This relationship, however, may change with altered lung conditions as airways may open or collapse during the SBT or after extubation. Therefore, whether the same threshold of 105 can be applied reliably to our data is questionable. However, during the SBT the RSBI was significantly higher at two time points in the failure group compared to the successfully weaned patients. Looking at EIT-derived changes from baseline may therefore provide more clinical guidance than a threshold value solely.

The transcutaneous CO_2_ increased in patients with a failed SBT. This could imply that SBT failure was mostly related to weak spontaneous breathing effort leading to carbon dioxide retention. Indeed, while the respiratory rate did not increase, minute ventilation decreased throughout the course of the SBT.

### Strengths and limitations

This was the first study aiming to perform long-term continuous EIT measurements (up to 24 h before and after extubation) during weaning, and where EIT measurements were combined with transcutaneous CO_2_ recordings. The reliability of long-term EIT measurements, however, posed an important challenge and could only be partially utilized in our analysis. To address this, we developed a novel combination of preprocessing techniques to filter out cardiovascular artifacts and identify stable tidal breathing periods, applicable to both ventilated and spontaneously breathing patients. Furthermore, we utilized the LuMon™ EIT device, featuring an oblique belt shape and lung contours for extraction of EIT parameters. The use of this device complements previous studies that used EIT devices from other manufacturers during the SBT, and also provides novel parameters (i.e., silent spaces) that were not previously investigated.

Our study had also several limitations. First, this observational study was performed in a small group with heterogeneous patients. Since EIT parameters during long term measurements around the weaning phase were unknown during the design of the study, a convenience sample was chosen. Although SBTs are protocolized in our ICU, differences were present in terms of the length of the SBT, mode and use of additional respiratory support after extubation, and also the flow of oxygen support during the SBT. Second, EIT signals are prone to several artifacts that could influence the reliability of parameters, including disturbances from alternating pressure relief mattresses which may have affected EELI calculations (but likely not other parameters) [29]. However, the effect was minimized by selection of stable tidal breathing periods.

Third, as mentioned earlier the reliability of comparing EIT parameters over a long time period was limited, for multiple reasons, including patient and belt movements, reapplication of contact agent on the EIT-belt (which was deemed necessary every several hours to obtain sufficient belt to skin contact), and noise and artifacts. The combination of these reasons affects the EIT signal and therefore reliable comparison of EIT parameters for prolonged recordings (supplemental figure A6). EIT researchers should be aware of these acquisition-related challenges when designing future studies with prolonged recordings. Therefore, recruitement/derecruitement after extubation could not be reliably assessed. As a result, we focused solely on EIT parameters directly around the SBT which were measured in a shorter and more stable period. Nevertheless, we explored the ventilation inhomogeneity for patients with a successful SBT and failed/successful extubation. These parameters are not affected by changes in EIT baseline and therefore can be adequately compared prior and after extubation.

### Clinical implications

Although the prediction of extubation failure is limited with EIT, EIT is a feasible non-invasive bedside measurement to obtain insight into the patient’s individual ventilation distribution and homogeneity of aeration. The ability to predict SBT failure based on EIT should be evaluated in further larger studies, and could have several clinical utilities. First, EIT is likely to be useful in signaling a patient’s ability to tolerate discontinuation of mechanical ventilation. If EIT parameters can predict SBT failure, they can help to identify patients at higher risk of failure early on. This allows clinicians to take appropriate actions promptly, adjust ventilation strategies, optimize respiratory support or consider alternative weaning approaches. Second, it facilitates individualized weaning strategies based on the underlying physiological factors identified by EIT that contribute to weaning difficulties. Third, it might help to avoid unnecessary extubation attempts which ensures patient safety and minimizes potential complications. Overall, the evaluation of EIT parameters during the SBT could support the clinicians’ decision-making process and might improve patient care during the weaning process from mechanical ventilation.

## Conclusions

Insight into physiological trends for the individual patient can be obtained with EIT during weaning from mechanical ventilation. Derecruitment after the SBT is common, observed by a drop in EELI without loss of tidal volume. The higher GI observed in patients failing to wean from mechanical ventilation can be attributed to regional differences in lung function. EIT has the potential to assess changes in ventilation distribution and quantify the inhomogeneity in electrical properties during the SBT, providing insights into the underlying physiological factors contributing to weaning failure.

### Supplementary Information

**Supplementary Material 1.**

## Data Availability

The datasets used and/or analysed during the current study are available from the corresponding author on reasonable request.

## Abbreviations

COVy: Center of Ventilation y-direction
COVx: Center of Ventilation x-direction
DSS: Dependent Silent Space
ΔZ: Delta Tidal Impedance
EIT: Electrical Impedance Tomography
EELI: End expiratory lung impedance
EILI: End inspiratory lung impedance
FLS: Functional Lung Space
GI: Global Inhomogeneity
ICU: Intensive Care Unit
MVeit: Minute Volume derived from EIT
NSS: Non-dependent Silent Space
PaCO_2_: Partial pressure of carbon dioxide
PaO_2_: Partial pressure of arterial oxygen
PEEP: Positive end-expiratory pressure
P/F: Partial pressure of oxygen to the fraction of inspired oxygen
RR: Respiratory Rate
RSBI_EIT_: Rapid Shallow Breathing Index derived from EIT
SAPS: Simplified Acute Physiology Score
SOFA: Sequential Organ Failure Assessment
SBT: Spontaneous Breathing Trial
TcCO_2_: Transcutaneous CO_2_
