# Supplementary material for: Electrical Impedance Tomography as a monitoring tool during weaning from mechanical ventilation: an observational study during the spontaneous breathing trial
Source: Respir Res. 2024 Apr 25;25:179. doi: 10.1186/s12931-024-02801-6 (PMC11044327; doi:10.1186/s12931-024-02801-6)
Supplement: Supplementary file 1 — Supplementary Material 1. [file 12931_2024_2801_MOESM1_ESM.docx]

**Appendix 1**

*Data pre-processing and analysis.*

An EIT preprocessing method to remove outliers, artefacts and noise components, including an automated selection of periods with stable tidal breathing was designed and applied in this paper. A detailed description of the three steps applied in this algorithm is described below:

1. Artefacts due to recalibration of the EIT signal (figure 1A) were identified as data points below zero and were removed from the signal.
2. Filtering of the cardiac artefacts was applied using a maximal overlap discrete wavelet transform (MODWT). The purpose of this filter is to remove impedance changes in the global and regional impedance distribution that are not caused by ventilation. By using the wavelet transform the EIT signal was decomposed into details and approximation parts in multiple levels, figure 1B. The lower levels correspond to high frequencies that predominantly consists of noise and cardiac components, whereas the higher levels are associated with low-frequency components and contain ventilation information. Characteristic for the wavelet transform is its capability to extract localized information. It has a very good trade-off between frequency and time information. As the heart rate is not fixed at a set frequency, the potential to extract localized information makes this MODWT filter encouraging.(1)We reconstructed the EIT signal with information from a subset of the wavelets: we used the symlet (sym4) wavelet with 5 levels of reconstruction. Only level 5 was used for reconstruction of the EIT signal.(2)
3. For EIT feature extraction it is important that the features are extracted from stable tidal breathing periods (STBP’s). A method proposed by Haris et al. was used for identification and analysis of stable breathing periods in EIT recordings, figure 1D. Three criteria were used for identification of STBP’s, namely the coefficient of variation of breath tidal volume, breath duration and the end-expiratory lung impedance (EELI).(3) A period is selected as an STBP as it fulfills the following requirements: it has a duration of 20 breaths and lasts for a minimum of 20 seconds. The following cutoff values were selected for the coefficients of variation: the CV_tidal-volume_ < 0.3, CV_breath-duration_ < 0.3 and CV_EELI_ < 0.3. The thresholds for the coefficient of variation are arbitrary, the lower the threshold the more periods are rejected as stable periods as they do not fulfill all requirements. A tradeoff is made here between the quality of the stable periods and the number of stable periods.

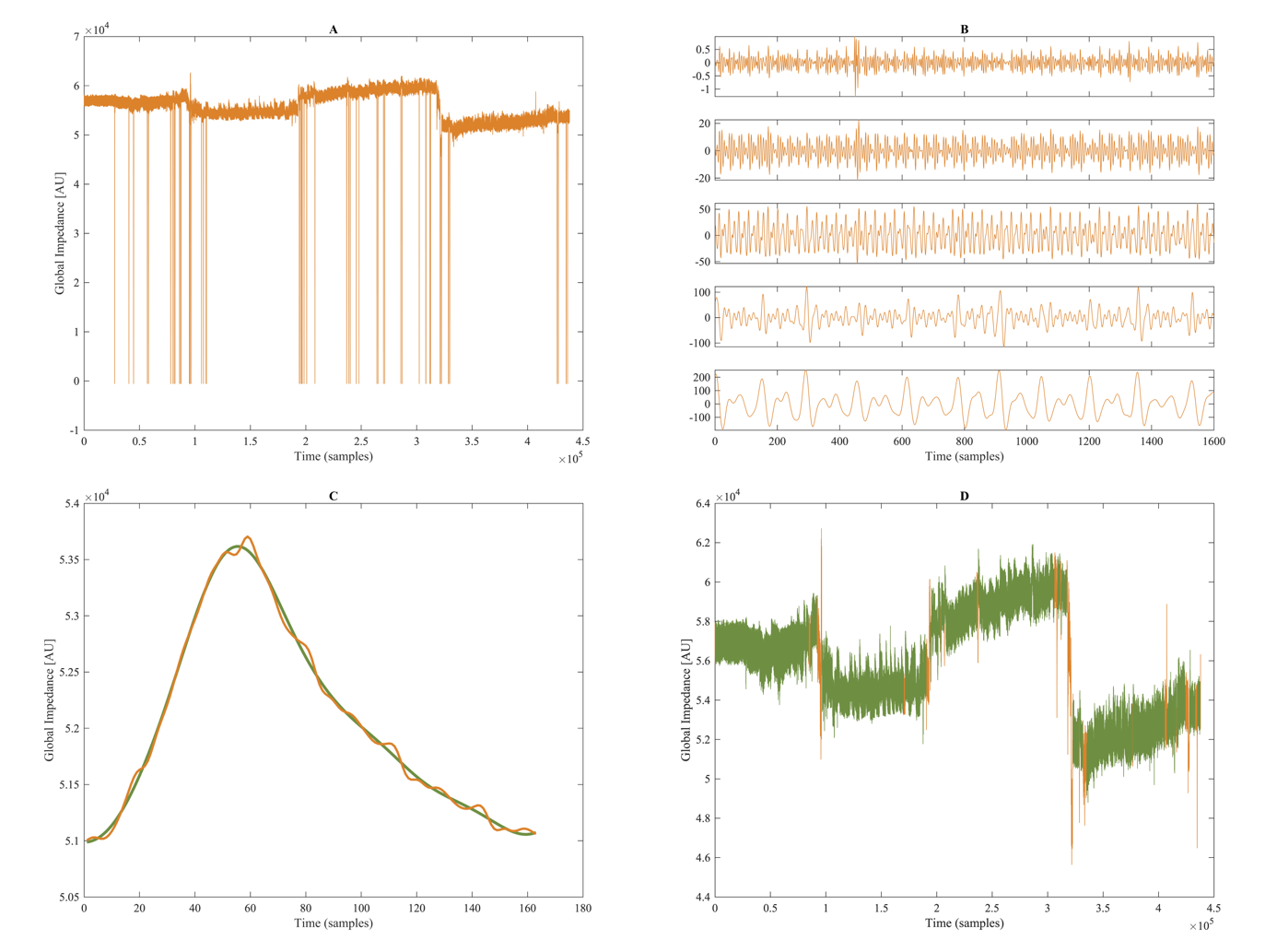

   **Figure A1 A.** Raw global impedance signal of a three-hour long EIT recording surround the spontaneous breathing trial and extubation from the mechanical ventilator. **B.** Reconstruction levels of the MODWT filter. The first upper levels contain the high frequencies and the bottom level contains the low frequency. **C.** Raw EIT signal of one breath in orange containing cardiac noise components. Filtered EIT signal (Green), noise components are removed without loss of ventilation information. **D.** Filtered EIT signal of the full three-hour recording were the STBPs are identified and marked green.

**References**

1. Addison PS. Wavelet transforms and the ECG: A review. Physiol Meas. 2005;26(5).

2. Daubechies I. Ten Lectures on Wavelets. Society for industrial and applied mathematics. Society for Industrial and Applied Mathematics; 1992.

3. Haris K, Vogt B, Strodthoff C, Pessoa D, Cheimariotis GA, Rocha B, et al. Identification and analysis of stable breathing periods in electrical impedance tomography recordings. Physiol Meas. 2021;42(6).

**Appendix 2**

***Figure A2:*** *Boxplots of EIT parameters during the SBT. None of these parameters showed a significant change (p > 0.05 for all) from baseline during the development of the SBT.*

**Appendix 3**

***Figure A3:*** *Boxplots of EIT parameters during the SBT with the non-successful SBT group in orange and the successful SBT group in green. None of these parameters showed a significant change from baseline nor a significant difference between the two groups during the development of the SBT (p > 0.05 for all).*

**Appendix 4**

***Figure A4:*** *Boxplots of EIT during the SBT. Patients that required reintubation (n=3) are orange and patients with successful extubation (n=18) are green. All patients had a successful SBT. None of these parameters showed a significant change from baseline nor a significant difference between the two groups during the development of the SBT (p > 0.05 for all).*

In our cohort 3 patients with a successful SBT required reintubation within 48 hours after extubation. Reasons for reintubation were: dypsnea, increased pCO_2_, insufficient oxygenation, increased breathing effort and exhaustion. We explored whether these patients show different behavior during the SBT compared to patients with successful extubation.

Figure A4 visualizes the results of a selection of parameters during the SBT for patients with a successful SBT and extubation and patients with a successful SBT and failed extubation. We observe a trend towards a stable transcutaneous CO_2_ in both groups, an elevated DSS for patients that were reintubated, and a slightly higher respiratory rate throughout the course of the SBT for the reintubated patients.  These results were not significantly different from the successfully extubated patients and should be interpretated with caution with only three patients requiring reintubation.

**Appendix 5**

***Figure A5:*** *Boxplots of EIT parameters around the SBT and after extubation. Patients that required reintubation (n=3) are orange and patients with successful extubation (n=18) are green. None of these parameters showed a significant change from baseline nor a significant difference between the two groups during the development of the SBT (p > 0.05 for all).*

**Appendix 6**

**
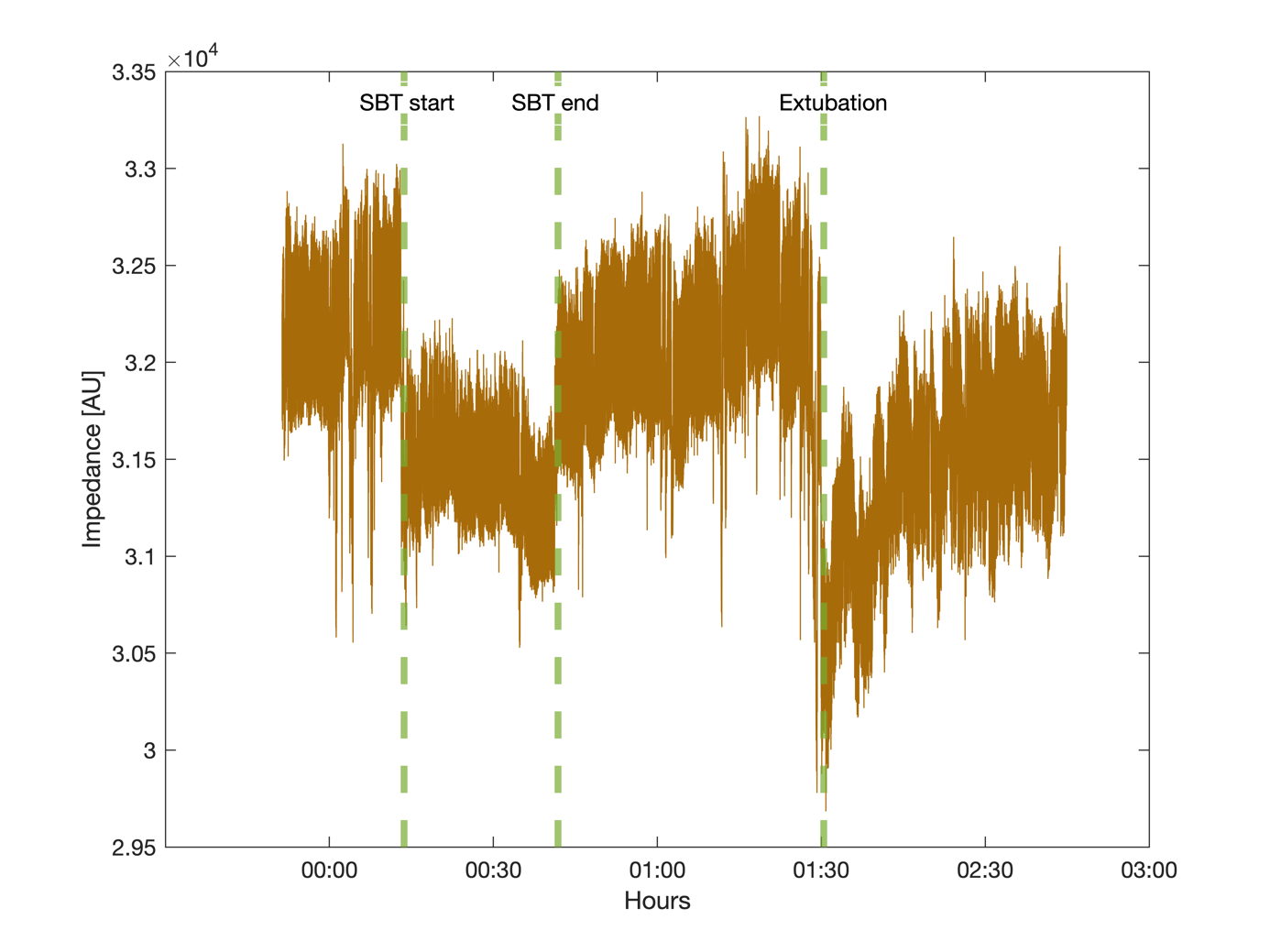
**

***Figure A6:*** *Global EIT signal to illustrate the variability in the impedance signal during prolonged recordings. Vertical lines indicate the start of the SBT, end of the SBT and extubation.*
